# Equivocal Dopamine Transporter Imaging: Integrating Visual Assessment, Quantification, and Clinical Follow-Up

**DOI:** 10.3390/life16091545

**Published:** 2026-09-16

**Authors:** So Young Kim

**Affiliations:** Department of Nuclear Medicine, Chung-Ang University Gwangmyeong Hospital, Chung-Ang University College of Medicine, 110 Deokan-ro, Gwangmyeong 14353, Gyeonggi-do, Republic of Korea; soykim@cauhs.or.kr

**Keywords:** dopamine transporter, FP-CIT, Parkinson disease, equivocal imaging, borderline scan, SWEDD, quantification, visual interpretation

## Abstract

Dopamine transporter (DAT) imaging is widely used to determine whether parkinsonian symptoms reflect presynaptic nigrostriatal degeneration. Although most examinations can be confidently classified as normal or abnormal, a clinically important subset remains equivocal. Such scans may show subtle posterior putaminal reduction, mild asymmetry, preserved comma-shaped morphology despite low quantitative values, or disagreement between visual and semiquantitative assessments. These findings may result from early neurodegeneration, physiological variation, medication effects, structural lesions, technical artifacts, or limitations of image processing and normative databases. This narrative review examines visual criteria, the complementary role and limitations of quantification, longitudinal outcomes of scans without evidence of dopaminergic deficit (SWEDD), common interpretive pitfalls, and the roles of clinical follow-up and repeat imaging. No universal quantitative cutoff can be applied across scanners, reconstruction methods, tracers, and software platforms. Expert visual interpretation remains the primary basis for assessment, while age- and platform-matched quantification is most useful as an adjunct in borderline cases. Integrating image quality, regional uptake pattern, quantitative findings, structural imaging, medication history, and clinical evolution may reduce diagnostic error and premature labeling of neurodegenerative parkinsonism.

## 1. Introduction

Parkinson disease (PD) is clinically defined by bradykinesia together with rest tremor, rigidity, or both [1,2]. Diagnostic confidence may nevertheless be limited in patients with mild or atypical symptoms, or in those whose symptoms have been modified by treatment [1,2]. Essential tremor, dystonic tremor, drug-induced parkinsonism, vascular parkinsonism, functional movement disorders, and several neurodegenerative syndromes may clinically overlap with PD [1,2,3,4]. Dopamine transporter imaging with single-photon emission computed tomography (SPECT) or positron emission tomography (PET) provides an in vivo marker of presynaptic nigrostriatal terminal integrity and is therefore useful when the clinical question is whether dopaminergic degeneration is present [3,4,5,6,7,8].

Typical neurodegenerative parkinsonism is characterized by reduced tracer binding in the posterior putamen, usually contralateral to the more affected side, with progressive extension to the anterior putamen and caudate nucleus [5,6,7,9]. In contrast, patients with essential tremor or pure drug-induced parkinsonism generally retain normal striatal binding [3,4,10,11]. International procedural guidance therefore emphasizes a visual assessment of striatal shape, intensity, and symmetry, supplemented by semiquantitative analysis when available [5,6]. However, the binary labels of normal and abnormal do not fully capture routine practice. Mild posterior putaminal thinning, subtle asymmetry, globally low uptake in an older patient, or a quantitative value close to the lower reference limit can preclude confident classification.

No standardized definition of equivocal DAT imaging has been established. Uncertainty may arise from poor technical quality, subtle visual abnormalities, disagreement between readers, discordance between visual and quantitative results, or a clinically convincing parkinsonian syndrome despite apparently preserved DAT binding. These situations matter because an abnormal result can reinforce a diagnosis of neurodegenerative parkinsonism and influence treatment decisions, whereas a normal result may prompt reconsideration of the diagnosis. Overcalling a borderline scan may give a patient an incorrect chronic disease label, while undercalling early degeneration may delay appropriate follow-up.

For the purposes of this review, we operationally define an equivocal DAT scan as a technically adequate examination that cannot be confidently classified as either normal or abnormal after standardized visual assessment. Such examinations typically show one or more borderline features, including mild posterior putaminal reduction or asymmetry without definite loss of normal striatal morphology, a regional quantitative value near the platform-specific reference boundary, or discordance among visual findings, quantitative measurements, and the clinical presentation.

Several broad reviews have summarized DAT radiotracers, imaging procedures, diagnostic performance, and clinical applications [1,2,3,4,7,8]. This review addresses a narrower practical issue: how should physicians interpret and manage a DAT examination that is neither clearly normal nor clearly abnormal? We focus on the integration of visual assessment, quantification, technical and biological confounders, SWEDD outcomes, and longitudinal clinical information.

For this narrative review, we conducted a focused literature search of PubMed/MEDLINE and Scopus for relevant publications available through August 2026. Search terms included combinations of “dopamine transporter imaging,” “DAT SPECT,” and “FP-CIT” with terms such as “equivocal,” “borderline,” “indeterminate,” “uncertain,” “discordant,” “SWEDD,” “semiquantification,” and “visual assessment.” English-language original articles, reviews, consensus statements, and practice guidelines relevant to the interpretation of equivocal or discordant dopamine transporter imaging were considered. Studies unrelated to presynaptic dopaminergic imaging and those lacking clinically relevant information on image interpretation were excluded. Reference lists of key articles and practice guidelines were also screened to identify additional relevant publications.

## 2. What Constitutes an Equivocal DAT Scan?

### 2.1. A Continuum Rather than a Binary State

DAT binding exists on a biological continuum, whereas clinical reports usually require a categorical conclusion. A scan can be considered clearly normal when bilateral caudate and putaminal uptake is intense and symmetric, with preservation of the expected comma-shaped configuration. A clearly abnormal scan shows definite loss of posterior putaminal uptake, distortion or shortening of the putaminal tail, marked asymmetry, or advanced reduction involving the entire putamen and caudate. Equivocal examinations fall between these two categories.

Common borderline appearances include mild, unilateral, posterior putaminal reduction without convincing shape distortion; symmetric but globally reduced striatal activity; preserved visual morphology with a quantitative Z-score near the lower limit; and apparent asymmetry that disappears after head reorientation [5,6,7,12,13,14,15,16,17,18]. Because motor manifestations generally emerge only after substantial nigrostriatal dysfunction has developed, a barely perceptible reduction in a patient with established typical PD should prompt a careful review of technical quality, clinical phenotype, and alternative diagnoses rather than automatic classification as positive [19,20,21].

### 2.2. Sources of Uncertainty

Uncertainty can arise at four levels. First, biological overlap exists between normal aging and early disease [14,22,23,24,25,26,27]. Second, acquisition and reconstruction affect image contrast and the apparent dimensions of the striatum [5,6,17,18]. Third, image processing may introduce registration or volume-of-interest errors [12,13,14,17,18,28]. Fourth, clinical uncertainty may persist even when imaging is technically adequate. These sources should be distinguished because quantification may help characterize subtle biological changes but cannot compensate for severe motion or failed spatial normalization [5,6,17,18,28].

## 3. Visual Interpretation

### 3.1. Stepwise Reading

Visual interpretation remains the primary approach recommended by international guidance [5,6]. A practical reading sequence begins with an assessment of image quality and head position, followed by an evaluation of background activity, localization of the caudate nuclei and putamina, side-to-side comparison, and inspection of the posterior putaminal tails [5,6]. The decisive feature is not reduced intensity alone but rather a pattern compatible with presynaptic degeneration: preferential posterior putaminal loss, an abnormal rostrocaudal gradient, and asymmetry concordant with the clinical laterality [5,6,7,9,10].

Physiological aging reduces DAT availability, but the reduction is usually gradual, bilateral, and shape-preserving [14,22,23,24,25,26,27]. Mild side-to-side differences can also occur in healthy individuals [14,26,27]. Therefore, mildly reduced overall intensity or small asymmetry without posterior predominance should not automatically be interpreted as evidence of neurodegeneration. Display normalization is another important source of variability [5,6]. Excessively narrow or wide color scales may exaggerate or conceal subtle deficits, and standardized display conditions should be used whenever possible [5,6].

### 3.2. Patterns Suggesting True Abnormality

Features that increase confidence in an abnormal interpretation include reproducible focal loss in the posterior putamen across adjacent slices, loss of the normal putaminal tail, reduction contralateral to the motor symptoms, and a concordant quantitative abnormality in the same subregion [5,6,7,9,10,12,13,14,15,16]. In advanced disease, reduced uptake may extend anteriorly and involve the caudate [9]. DAT imaging confirms presynaptic dopaminergic degeneration but does not reliably distinguish PD from multiple system atrophy, progressive supranuclear palsy, corticobasal degeneration, or dementia with Lewy bodies [1,2,29,30].

### 3.3. Patterns Favoring a Normal or Non-Degenerative Result

Preserved bilateral putaminal tails and symmetric comma-shaped uptake favor a normal result, even when overall striatal uptake appears mildly reduced [5,6,7,10]. Normal DAT imaging is expected in essential tremor, many cases of drug-induced parkinsonism, dystonic tremor, and functional parkinsonism [3,4,10,11,31,32]. Vascular parkinsonism is heterogeneous: DAT binding may be preserved when the nigrostriatal terminals are intact, whereas a focal infarction involving the striatum can produce a localized defect [30,33,34]. A review of magnetic resonance imaging (MRI) or computed tomography (CT) is therefore essential when uptake loss does not follow the characteristic posterior putaminal pattern [11,30,33,34].

### 3.4. Inter-Reader Variability and Reader Experience

Although visual interpretation generally shows good interobserver agreement for clearly normal and clearly abnormal examinations, agreement may be lower for subtle regional abnormalities and borderline uptake patterns [35,36]. Reader experience is therefore particularly relevant in equivocal cases. Semiquantitative information may increase diagnostic confidence and can be particularly helpful for readers with limited experience [36]. When the distinction between normal variation and subtle dopaminergic loss remains uncertain, review by a second experienced reader together with regional quantitative assessment should therefore be considered.

## 4. Quantitative Analysis in Borderline Cases

### 4.1. Metrics and Their Intended Role

Semiquantitative analysis commonly expresses striatal binding relative to a low-DAT reference region [7,8,12,13,14,15,16]. SPECT software often reports specific binding ratios, whereas static PET studies commonly use standardized uptake value ratios [7,8,12,13,14,15,16]. Regional measures may include the whole striatum, caudate, anterior and posterior putamen, putamen-to-caudate gradients, and asymmetry indices [7,8,12,13,14,15,16]. Quantification is particularly useful when visual findings are subtle, but it should be regarded as an adjunct rather than a stand-alone diagnostic test [5,6,7,12,13,14,15,16].

The key quantitative question is whether the region that appears subtly abnormal lies outside an age-appropriate reference distribution [12,13,14,15,16,17,18]. A posterior putaminal Z-score below the software-defined normal range can strengthen suspicion when the visual pattern is concordant [12,13,14,15,16,17,18]. Conversely, normal regional values may reduce the likelihood that minor visual asymmetry represents neurodegeneration [12,13,14,15,16,17,18]. Nevertheless, a single borderline Z-score should not outweigh a technically sound expert visual assessment, particularly when image registration is imperfect [5,6,12,13,14,15,16,17,18].

### 4.2. Why There Is No Universal Cutoff

A universal cutoff cannot be safely transferred between institutions because measured binding depends on the tracer, scanner resolution, acquisition timing, reconstruction, filtering, attenuation correction, spatial normalization, volume-of-interest definition, and reference region [5,6,7,12,13,14,15,16,17,18]. PET values may be particularly sensitive to reconstruction and resolution recovery [7,15,16,17,18]. Software platforms also use different normative cohorts and algorithms [12,13,14,15,16,17,18]. Accordingly, published absolute cutoff values should not be applied directly unless the local protocol has been validated using the same acquisition and processing framework [5,6,7,12,13,14,15,16,17,18].

Ideally, a normal database should be acquired with the same scanner and reconstruction used in clinical practice [5,6,7,12,13,14,15,16,17,18]. In reality, most centers rely on a manufacturer- or software-provided database. Local validation should confirm accurate classification of clearly normal and clearly abnormal cases, assess registration failures, and account for age and sex effects [12,13,14,17,18,25,26,27]. Harmonization approaches may improve multicenter comparability, but they do not eliminate the need for quality control [12,13,14,15,16,17,18].

A practical local validation process can be performed stepwise. First, the center should define and maintain a standardized acquisition, reconstruction, and processing protocol for which the normative database will be used. Second, the demographic characteristics, tracer, scanner configuration, reconstruction method, and analysis procedure of the software-provided reference database should be reviewed for compatibility with the local population and protocol. Third, regional values should be examined in locally available subjects with visually normal, non-degenerative DAT patterns across an appropriate age range, with particular attention to age-related decline and, where relevant, sex effects. Where sufficient local reference data are available, statistical validation should assess whether age-adjusted regional Z-scores are centered around the expected reference value and whether residual age dependence remains after age correction, for example, using regression analysis. Statistically and clinically meaningful site-specific offsets or residual age-related trends should prompt local recalibration of age-matched thresholds [14,25,37]. Fourth, the software should be tested using representative clearly normal and clearly abnormal examinations to identify systematic bias, registration failure, segmentation error, or discordance with expert visual interpretation. Finally, validation should be repeated after substantial changes in scanner hardware, reconstruction, software version, or processing parameters. When available, phantom-based or statistical harmonization can further support comparability across scanners [37], but it does not replace clinical validation of the normative database.

### 4.3. Visual–Quantitative Discordance

When visual and quantitative results disagree, the discrepancy should be investigated rather than resolved by default in favor of one method [5,6,7,12,13,14,15,16,17,18]. A visually abnormal scan with normal quantification may reflect early focal posterior loss diluted within a larger volume of interest, database mismatch, or reader overinterpretation [12,13,14,15,16,17,18]. Conversely, abnormal quantitative values with visually preserved uptake may result from global intensity normalization, age mismatch, imperfect registration, partial-volume effects, or medication-related reduction [12,13,14,15,16,17,18,38,39,40,41]. The clinically relevant question is whether both methods identify the same anatomically plausible abnormality [12,13,14,15,16,17,18,28,39,40].

### 4.4. Multivariate Analysis

Beyond conventional univariate quantitative measures, multivariate approaches may provide additional value in equivocal cases. Models incorporating regional DAT binding, interhemispheric asymmetry, caudate-to-putamen gradients, age, and MRI-derived measures of striatal or broader brain morphology may capture complementary aspects of neurodegeneration that are not represented by a single region-of-interest (ROI) cutoff. Studies combining DAT SPECT with structural and diffusion MRI, including recent multimodal machine-learning approaches, support the complementary value of these modalities for characterizing disease severity and progression [42,43]. Such approaches may be particularly useful when mild functional abnormalities coexist with subtle structural changes. However, their added diagnostic value specifically for equivocal DAT scans remains to be established, and external validation across scanners, software platforms, and patient populations is required before routine clinical implementation.

## 5. Technical and Biological Pitfalls

### 5.1. Motion, Head Rotation, and Reconstruction

Patient motion blurs the striatum and may reduce apparent uptake or create asymmetry [5,6]. Head rotation changes the axial appearance of the putamen and can mimic shortening of one posterior tail [5,6]. Reorientation to a standard plane and review of projection data or dynamic frames, when available, are essential [5,6]. Reconstruction parameters affect resolution, contrast, and measured ratios [17,18]; therefore, follow-up examinations should ideally use the same protocol and software [5,6,17,18].

### 5.2. Structural Lesions and Atrophy

Infarction, hemorrhage, tumor, prior surgery, and marked basal ganglia atrophy can produce focal or regional reductions unrelated to an underlying neurodegenerative parkinsonian syndrome [11,30,33,34,39,40]. When a defect conforms to a vascular territory or known structural lesion, it should be interpreted in conjunction with MRI or CT [11,30,33,34]. Atrophy, volume-of-interest contamination, and partial-volume effects may alter measured uptake and binding ratios, particularly in older individuals and patients with advanced neurodegeneration [39,40].

Structural MRI can be particularly helpful when visual and quantitative DAT findings are discordant. Because limited SPECT resolution and striatal atrophy may affect measured binding through partial-volume effects, accurate MRI coregistration can improve anatomical localization and, when available, support more precise volume-of-interest (VOI) definition and partial-volume correction [44]. However, these methods are not yet standardized and should be considered adjunctive rather than mandatory for routine DAT interpretation.

When equivocal DAT findings persist after consideration of structural lesions and partial-volume effects, additional MRI biomarkers may provide complementary information. Neuromelanin-sensitive MRI and iron-sensitive techniques, including susceptibility-weighted imaging and quantitative susceptibility mapping, can characterize substantia nigra integrity from complementary tissue-sensitive perspectives. Reduced neuromelanin signal or volume and increased nigral susceptibility reflecting iron accumulation have been reported in PD and may provide additional information when DAT findings are borderline or discordant [45,46]. However, diagnostic performance varies with disease stage and methodology, acquisition and analysis remain heterogeneous, and these MRI biomarkers have not been established as definitive adjudication tools for equivocal DAT scans [45,46].

### 5.3. Tracer-Related Considerations and Emerging DAT Radiotracers

Several DAT radiotracers have been developed for SPECT and PET imaging. Although ^123^I-fluoropropyl-carbomethoxy-iodophenyl-nortropane (^123^I-FP-CIT) remains the most widely established SPECT ligand, PET tracers offer higher spatial resolution and greater quantitative potential. ^18^F-FP-CIT has established clinical use in some regions, whereas ^11^C-PE2I provides high DAT selectivity but is constrained by the short half-life of carbon-11 [7]. More recently, ^18^F-FE-PE2I has shown favorable kinetics, high selectivity, and feasibility of short static acquisitions, with diagnostic performance comparable to ^123^I-FP-CIT SPECT [47]. ^18^F-LBT-999 is another highly selective fluorinated DAT ligand with clinical feasibility demonstrated using short static imaging windows [48]. Importantly, recent data suggest that ^18^F-FE-PE2I PET may provide additional clarification in selected patients with persistent diagnostic uncertainty after abnormal ^123^I-FP-CIT SPECT, although its specific role in equivocal DAT imaging requires further validation [49]. Because tracer kinetics, acquisition protocols, and normative databases differ, quantitative thresholds should not be transferred directly between tracers [5,6,7,12,13,14,15,16,17,18].

### 5.4. Medication Effects

DAT-affinity drugs may reduce tracer binding and mimic dopaminergic loss [5,6,38]. Cocaine, amphetamine derivatives, methylphenidate, modafinil, and bupropion are among the drugs of greatest concern [5,6,38]. Selective serotonin reuptake inhibitors may have smaller or tracer-dependent effects [38,41]. By contrast, levodopa and most dopamine agonists generally do not require withdrawal solely for DAT imaging, although local product information and procedural guidance should be followed [5,6,38]. Medication history is particularly important when visual findings are preserved but quantitative values are unexpectedly low [5,6,38,41]. When clinically safe and appropriate, medications or substances with substantial DAT affinity should be withheld for at least five elimination half-lives before tracer administration, or according to a longer drug-specific interval when recommended. The agent, time of the last dose, and actual washout interval should be documented; when withdrawal is incomplete or not feasible, the potential pharmacological effect should be explicitly considered during interpretation [5,6,38].

### 5.5. Normal Aging and Biological Variation

DAT availability declines with age, and sex-related differences have also been reported [14,22,23,24,25,26,27]. This age-related decline does not reproduce the posterior-predominant pattern typical of PD [9,14,22,23,24,25,26,27]. Age-corrected reference data are therefore preferable to fixed absolute thresholds [14,25,26,27]. Ethnic background, handedness, and individual asymmetry may contribute additional variation, although their clinical effects are generally smaller than those of age, technical factors, and disease [14,22,23,24,25,26,27]. Beyond physiological variation, early neurodegenerative changes may also contribute to borderline DAT findings before overt structural degeneration becomes apparent. Mitochondrial homeostasis dysregulation, impaired cellular quality-control mechanisms, and coexisting proteinopathies may increase neuronal and synaptic vulnerability and contribute to heterogeneous trajectories of functional decline. For example, interactions between TDP-43 pathology and mitochondrial dysfunction have been implicated in neuronal vulnerability in Alzheimer disease [50]. Although these mechanisms do not directly explain equivocal DAT findings, they illustrate how cellular dysfunction may precede macroscopic structural changes and contribute to intermediate imaging phenotypes. Emerging precision genome-engineering technologies may provide experimental models to further investigate these early mitochondrial and proteostatic abnormalities, although their relevance to DAT-imaging phenotypes remains investigational [51]. Table 1 summarizes common causes of equivocal DAT findings and the corresponding practical responses.

### 5.6. Prodromal Phenotypes and Complementary Imaging

Biological heterogeneity may also contribute to variable DAT imaging phenotypes. The proposed brain-first/body-first model suggests that α-synuclein pathology may begin predominantly in central or peripheral autonomic structures, respectively, with isolated REM sleep behavior disorder (iRBD), constipation, and autonomic dysfunction more often associated with the proposed body-first phenotype [52]. Although earlier models suggested relatively symmetric nigrostriatal degeneration in body-first PD and greater asymmetry in brain-first PD, recent DAT-SPECT studies have not consistently confirmed this distinction, and conventional DAT imaging alone cannot currently classify these phenotypes [53,54]. In prodromal populations, reduced striatal DAT binding can identify subgroups at increased risk of phenoconversion among individuals with hyposmia or iRBD, although these findings are not sufficiently specific for individual diagnosis [55,56].

Cardiac ^123^I-metaiodobenzylguanidine (MIBG) scintigraphy may provide complementary information in equivocal DAT cases by assessing postganglionic sympathetic denervation. Reduced myocardial MIBG uptake is frequently observed in PD and can support a Lewy body-related diagnosis, although normal uptake does not exclude PD and cardiac or medication confounders must be considered [57]. Reduced cardiac MIBG uptake has also been associated with iRBD and proposed body-first phenotypes [58,59]. Gastrointestinal ^11^C-donepezil PET studies have reported reduced small-intestinal and colonic cholinergic uptake in early PD and more prominent autonomic abnormalities in iRBD and PD with REM sleep behavior disorder (RBD) [59,60]. However, intestinal cholinergic PET remains investigational, and neither modality has been specifically validated as a routine adjudication test for equivocal DAT imaging.

## 6. SWEDD and Longitudinal Outcomes

### 6.1. SWEDD Is a Descriptor, Not a Diagnosis

SWEDD—scans without evidence of dopaminergic deficit—was introduced to describe participants clinically diagnosed with early PD whose DAT imaging remained within the normal range [31,61,62,63,64]. It is a descriptive category rather than a single disease entity [31,63,64,65,66]. Longitudinal studies have shown that many participants classified as SWEDD display minimal imaging progression [61,63,64] and are later assigned alternative diagnoses, such as essential or dystonic tremor, functional movement disorder, or other non-degenerative conditions [31,32,63,64,66].

In the PRECEPT cohort, participants with SWEDD showed little change in striatal binding over approximately 22 months and less clinical progression than those with imaging-confirmed PD, suggesting that most were unlikely to have typical idiopathic PD [61]. PPMI likewise identified participants with normal DAT imaging despite a clinical diagnosis of early PD, underscoring the limitations of clinical diagnosis at very early stages [62]. These findings indicate that preserved DAT binding should not automatically be dismissed as a false-negative scan in every patient clinically labeled as having PD.

### 6.2. Can a Normal Scan Become Abnormal?

Conversion from visually normal to abnormal DAT imaging does occur, but reported rates vary markedly with patient selection, follow-up duration, baseline image quality, and the definition of SWEDD [31,63,64,65,66]. Small studies of broadly defined SWEDD populations have generally found that most scans remain normal [31,61,63,64], whereas selected cohorts of patients with characteristic rest tremor and rigidity have reported higher conversion rates [63,65,66]. Some apparent conversions may reflect borderline abnormalities already present at baseline, better image quality at follow-up, or differences in interpretation rather than true biological conversion [31,63,64,65,66].

A normal baseline scan therefore does not exclude the possibility of future degeneration [31,63,64,65,66], but routine repeat imaging is not justified for every patient classified as SWEDD [31,61,63,64,65,66]. Repeat DAT imaging is reasonable when the clinical syndrome develops clear bradykinesia and rigidity, symptoms progress despite an initially normal scan, or retrospective review identifies subtle posterior putaminal reduction [5,6,31,63,64,65,66]. The interval should be determined by the clinical course rather than by a fixed schedule [5,6,31,63,64,65,66].

### 6.3. Levodopa Response Does Not Resolve Imaging Uncertainty

A clinical response to levodopa indicates that symptoms are dopamine responsive, but it is not specific for idiopathic PD [1,32,67]; lack of response may reflect an inadequate dose or treatment duration, poor absorption, or atypical pathology [1,67]. DAT imaging and levodopa response assess related but distinct aspects of the motor system [1,32,67]. Abnormal DAT binding indicates presynaptic terminal loss but does not reliably predict the magnitude of levodopa response, because atypical neurodegenerative parkinsonian syndromes may also show severe DAT reduction with limited treatment benefit [1,2]. Conversely, rare disorders such as dopa-responsive dystonia may show preserved DAT imaging despite a marked response to levodopa [67].

### 6.4. Clinical Follow-Up and Repeat Imaging

A practical approach to an equivocal DAT scan should begin with a reassessment of technical quality, medication effects, structural lesions, and the concordance between visual and quantitative findings. If uncertainty remains but the clinical phenotype is stable or only weakly suggestive of neurodegenerative parkinsonism, continued clinical observation is generally preferable to immediate repeat imaging. Repeat DAT imaging may be considered when clinically meaningful progression occurs, such as new or progressive bradykinesia, rigidity, increasing asymmetry, gait impairment, or other features that strengthen suspicion of a neurodegenerative parkinsonian syndrome, particularly when the initial scan was genuinely borderline and the result would alter management. When repeat imaging is considered necessary, an interval of approximately 12 months may be reasonable, although the optimal timing has not been firmly established [68]. In contrast, routine repeat imaging is generally unnecessary after a clearly normal or characteristically abnormal scan. Thus, the decision to repeat DAT imaging should be driven primarily by persistent clinical–imaging discordance and interval phenotypic evolution rather than by a fixed imaging schedule.

## 7. A Practical Clinical Decision Framework

Interpretation of an equivocal examination should begin by identifying the source of uncertainty [5,6]. If technical quality is inadequate, the study should be reported as technically limited rather than given a biological classification [5,6,17,18]. If image quality is acceptable, the reader should determine whether the suspected abnormality follows a disease-compatible posterior putaminal pattern [5,6,7,9,10]. Quantitative results should then be reviewed region by region, with attention to age correction, registration, reference region, and platform-specific limits [12,13,14,15,16,17,18,25,26,27]. Structural imaging and medication history should be considered before a final conclusion is reached [5,6,11,30,33,34,38,39,40,41].

Indeterminate reporting language is appropriate when the available findings remain inconclusive. Suggested wording is: “Mild asymmetric reduction in posterior putaminal uptake is present; however, the visual findings are insufficient to establish a presynaptic dopaminergic deficit. Quantitative values are [within/below] the platform-specific reference range. Clinical follow-up and correlation with medication history and structural imaging are recommended.” This wording states the uncertainty directly without classifying the scan as either normal or abnormal.

**Figure 1:**
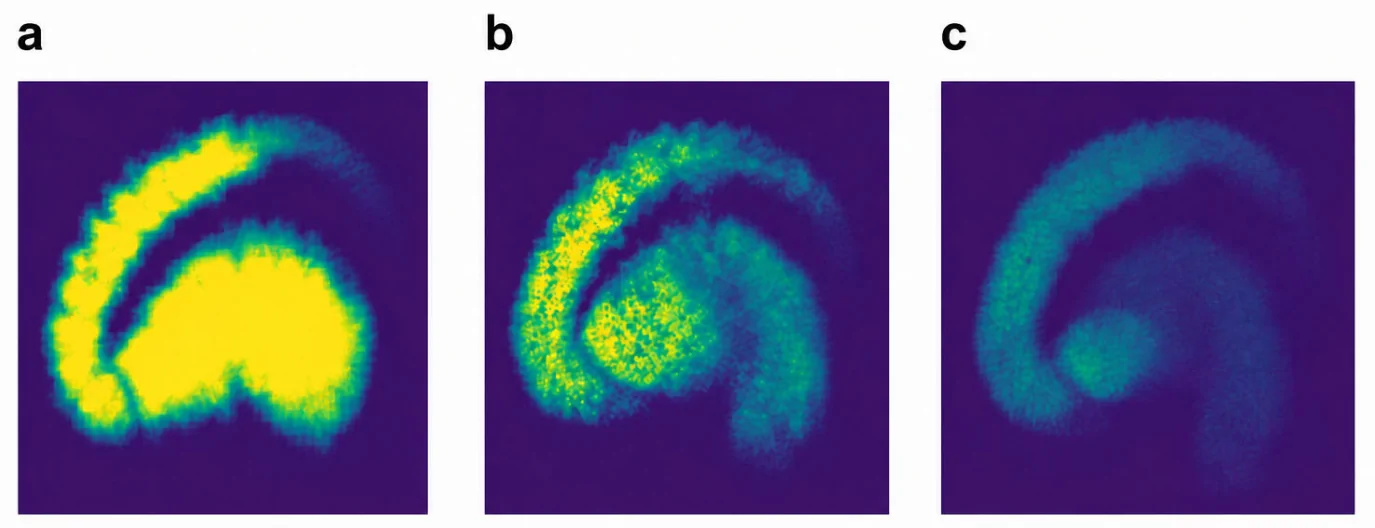
Conceptual spectrum of dopamine transporter imaging findings. Illustrative images show (**a**) a clearly normal pattern with preserved striatal uptake, (**b**) an equivocal pattern with mildly reduced uptake, and (**c**) a clearly abnormal pattern with markedly reduced striatal uptake. The panels are intended to illustrate the diagnostic continuum and are not intended for quantitative comparison. Abbreviation: DAT, dopamine transporter.

Management should be guided by the overall clinical phenotype [1,5,6]. A clearly normal scan of a patient with isolated action tremor should shift attention toward a non-degenerative diagnosis [3,4,10,31]. An equivocal scan of a patient with progressive asymmetric bradykinesia and rigidity warrants review by an experienced reader, quantitative reassessment, and neurological follow-up [5,6,12,13,14,15,16,17,18]. Repeat imaging may be considered when the result would alter management and after an interval sufficient for a detectable biological change [5,6,31,63,64,65,66]. DAT imaging should not be used as a screening test in clinically typical, established PD, nor should serial imaging be performed solely to document progression in routine care [1,5,6]. The spectrum of findings is illustrated in Figure 1.

## 8. Artificial Intelligence and Future Directions

Automated segmentation, deep learning-based spatial normalization, and artificial intelligence (AI)-based classification algorithms may reduce reader variability and improve detection of subtle regional deficits [28,69,70,71,72,73,74,75]. Although several systems have shown high diagnostic performance in selected datasets [28,69,70,71,72,73,74,75], performance may decline when they are applied to different scanners, reconstruction methods, tracers, or populations [37,73,74,75]. In the near term, the most useful roles of AI are likely to be quality control, objective regional measurement, and identification of visual–quantitative discordance rather than autonomous diagnosis [28,69,70,71,72].

Future studies should define equivocal imaging prospectively, use expert consensus and clinical diagnosis established during follow-up as reference standards, and report region-specific quantitative distributions rather than a single whole-striatum cutoff [12,13,14,15,16,17,18,31,63,64,65,66]. For AI-based interpretation, domain shift and out-of-distribution data remain important challenges because differences in scanners, acquisition and reconstruction protocols, preprocessing, tracers, and patient populations may reduce performance outside the training environment [37,73,74,75]. Multicenter harmonization, heterogeneous training datasets, and independent external and prospective validation are therefore needed before quantitative thresholds or AI outputs can be generalized [14,28,37,69,70,71,72,73,74,75]. Studies should also distinguish truly normal baseline scans from retrospectively recognizable borderline abnormalities in patients whose follow-up scans later become abnormal [31,63,64,65,66].

## 9. Conclusions

Equivocal DAT imaging is a clinically meaningful problem that cannot be resolved by a single visual sign or universal quantitative cutoff. Expert visual assessment remains the foundation of interpretation, with particular emphasis on the posterior putaminal pattern, morphology, and asymmetry. Quantification is most valuable as an adjunct when visual findings are subtle, provided that age- and platform-matched normative data, registration quality, and technical factors are considered. Most patients classified as SWEDD do not show the imaging progression expected in typical PD, although selected patients may later develop abnormal scans. A structured assessment of technical quality, regional uptake pattern, quantitative results, medication history, structural imaging, and longitudinal clinical evolution provides a practical approach to this diagnostic gray zone.

## Figures and Tables

**Table 1:** Common causes of equivocal DAT findings and practical responses.

Cause	Potential Appearance	Practical Response
Motion	Blurred or asymmetric striata	Inspect raw frames; repeat the acquisition or reconstruct with motion correction when feasible.
Head rotation	Apparent unilateral posterior shortening	Reorient images in three planes before interpretation.
Normal aging	Symmetric global reduction	Use age-matched normative data; focus on pattern and shape.
DAT-binding medication	Diffuse or regional quantitative reduction	Review the medication list and last dose; when clinically safe, withhold relevant interfering agents for ≥5 elimination half-lives or the recommended drug-specific interval.
Striatal infarct/lesion	Focal defect not typical of PD	Correlate with MRI/CT.
Atrophy/partial-volume effect	Reduced measured uptake	Review structural imaging and verify segmentation accuracy.
Database mismatch	Unexpected abnormal Z score	Use platform-specific norms and local validation.
Early degeneration	Subtle posterior putaminal loss	Seek concordance among uptake pattern, quantitative results, symptoms, and follow-up.

Abbreviations: CT, computed tomography; DAT, dopamine transporter; MRI, magnetic resonance imaging; PD, Parkinson disease.

## Data Availability

No new data were created or analyzed in this study. Data sharing is not applicable to this article.

## Abbreviations

AI, artificial intelligence; CT, computed tomography; DAT, dopamine transporter; FP-CIT, fluoropropyl-carbomethoxy-iodophenyl-nortropane; iRBD, isolated REM sleep behavior disorder; MIBG, metaiodobenzylguanidine; MRI, magnetic resonance imaging; PD, Parkinson disease; PET, positron emission tomography; RBD, REM sleep behavior disorder; ROI, region of interest; SPECT, single-photon emission computed tomography; SWEDD, scans without evidence of dopaminergic deficit; VOI, volume of interest.
